# Micropeptides: potential treatment strategies for cancer

**DOI:** 10.1186/s12935-024-03281-w

**Published:** 2024-04-15

**Authors:** He Zhou, Yan Wu, Ji Cai, Dan Zhang, Dongfeng Lan, Xiaofang Dai, Songpo Liu, Tao Song, Xianyao Wang, Qinghong Kong, Zhixu He, Jun Tan, Jidong Zhang

**Affiliations:** 1 Department of Immunology, Zunyi Medical University, Zunyi City, Guizhou Province 563000 China; 2 Special Key Laboratory of Gene Detection & Therapy of Guizhou Province, Zunyi Medical University, Zunyi, 563000 China; 3 Zunyi Medical University Library, Zunyi, 563000 China; 4 Guizhou Provincial College-based Key Lab for Tumor Prevention and Treatment with Distinctive Medicines, Zunyi Medical University, Zunyi563000, China; 5 Collaborative Innovation Center of Tissue Damage Repair and Regeneration Medicine, Zunyi Medical University, Zunyi, 563000 China; 6 Department of Histology and Embryology, Zunyi Medical University, Zunyi, 563000 China

**Keywords:** Micropeptide, Subcellular localization, Cancer, Noncoding RNAs

## Abstract

Some noncoding RNAs (ncRNAs) carry open reading frames (ORFs) that can be translated into micropeptides, although noncoding RNAs (ncRNAs) have been previously assumed to constitute a class of RNA transcripts without coding capacity. Furthermore, recent studies have revealed that ncRNA-derived micropeptides exhibit regulatory functions in the development of many tumours. Although some of these micropeptides inhibit tumour growth, others promote it. Understanding the role of ncRNA-encoded micropeptides in cancer poses new challenges for cancer research, but also offers promising prospects for cancer therapy. In this review, we summarize the types of ncRNAs that can encode micropeptides, highlighting recent technical developments that have made it easier to research micropeptides, such as ribosome analysis, mass spectrometry, bioinformatics methods, and CRISPR/Cas9. Furthermore, based on the distribution of micropeptides in different subcellular locations, we explain the biological functions of micropeptides in different human cancers and discuss their underestimated potential as diagnostic biomarkers and anticancer therapeutic targets in clinical applications, information that may contribute to the discovery and development of new micropeptide-based tools for early diagnosis and anticancer drug development.

## Introduction

A number of genetic abnormalities, epigenetic changes, chromosomal translocations, deletions, and amplifications have been linked to the highly complicated disease known as cancer [1].With the ageing of the population, the number of malignant tumour incidences and deaths continues to increase, and the global cancer burden continues to increase [2]. As a significant disease endangers human health, the earlier cancer is detected, the more effective the treatment can be, and the improved survival rate can be greatly improved. However, approximately one-half of all cancers are still detected only at a late stage. Despite recent advances in early cancer detection and the emergence of many promising markers for early detection, such as circulating tumour DNA, proteins, and exosomes, further innovation and development of early cancer detection methods are required [3]. Notably, with advances in ribosomal blotting and various histological analysis techniques, RNA, previously considered to be a noncoding nucleotide, is useful for early detection. RNAs previously annotated as incapable of encoding have also been identified as capable of encoding micropeptides with vital biological functions and are closely related to cancer development.

Many unique RNA transcripts known as noncoding RNAs (ncRNAs) have been discovered in eukaryotic genomes, and they are essential for numerous cellular and physiological processes [4]. These RNAs include long noncoding RNAs (lncRNAs), circular RNAs, microRNAs, and small nucleolar RNAs (snoRNAs) [5]. Small open reading frames (sORFs) are found to be abundant in the molecular sequences of ncRNAs. In previous studies, the majority of ORF finding algorithms set the minimum size for detection to 300 nucleotides or 100 amino acids, which resulted in certain small sORFs being undiscovered due to their short sequence length and limitations of research techniques [6]. The rapid development of histological analysis, ribosome analysis, and bioinformatics has revealed that some small open reading frames (sORFs) in ncRNAs show the potential to encode micropeptides.

Micropeptides are small peptides encoded by sORFs, are less than 100–150 amino acid sequences in length, and exhibit the advantages of small size, high specificity, and low cytotoxicity [7, 8]. sORF-encoded micropeptides play significant roles in tumour progression and are involved in various cellular processes, including signal transduction, calcium transport, mitochondrial regulation, DNA repair, transcriptional regulation, inflammatory immunity, and embryonic development [9]. Both tumour inducers and tumour suppressors are found among these micropeptides. Understanding the function of ncRNA-encoded micropeptides in cancer may lead to new perspectives on the clinical management and prognosis of the disease. In this review, we describe the sources of micropeptides, how they are identified, and the roles they play in various subcellular localizations in the context of cancer. Finally, we discuss the potential clinical value of these micropeptides and how they might be used in the detection and treatment of cancer. This information may be useful for the discovery and optimization of micropeptides based on cancer-specific target molecules for the assessment of cancer prognosis and the development of new therapeutic strategies.

## Sources of micropeptides

Using a combination of ribosome analysis, peptide labelling, and bioinformatics analysis, recent studies have reported noncoding transcripts containing sORFs with the potential to encode micropeptides, including long noncoding RNA (lncRNA), mRNA (5-upstream, coding structural domain and 3-downstream), circular RNA, and miRNA [10–12].

### LncRNAs

LncRNAs, mainly transcribed by RNA polymerase II (Pol II), are recognized as RNAs with a length of more than 200 nucleotides (nt) and play important roles in many physiological functions through a variety of mechanisms. To control chromosome shape and function, regulate gene transcription in cis or trans, and influence mRNA splicing, stabilization, and translation, they interact with a variety of components, including DNA, RNA, and proteins [13, 14]. Due to the lack of canonical open reading frames (ORFs > 100 aa), lncRNAs had been widely believed to be untranslatable. However, lncRNAs structures are comparable to mRNA structures; lncRNAs carry a poly(A) tail at the 3’ end and a 7-methylguanosine triphosphate (m7G-cap) at the 5’ end, suggesting that they may show translation activity similar to that of mRNA [15]. Researchers discovered that yeast ncRNAs carrying sORFs were more susceptible to nonsense-mediated RNA decay (NMD) by finding ncRNAs. The authors proposed that some ncRNAs in yeast also encode proteins because only RNAs that encode proteins are vulnerable to NMD [16]. Additionally, other evidence pointed to lncRNAs encoding proteins or small peptides with distinct biological activity and that sORFs initiate the translation of lncRNAs. Recently, with the identification of many micropeptides encoded by lncRNAs, an expanded view of the scope and action of lncRNAs has been recognized, with some have been found to be involved in metabolism, inflammation, and cancer.

### CircRNAs

CircRNAs constitute a class of circular RNA molecules transcribed by Pol II that lack a 5′ cap or 3’ poly(A) tail. Because of their circular structure, circRNAs are resistant to digestion by the exonuclease RNase, making them more stable than linear RNAs. CircRNAs undergo mRNA splicing and facilitate, protein location regulation, and transcription control [17, 18]. Numerous micropeptides encoded by circRNAs have been discovered, including circ-LINC-PINT, circ0000437, and circPPP1R12A. These micropeptides have been translated in vivo, and their functions have been identified and described as a result of cutting-edge deep sequencing technologies and computational methods [19–21].

Recent studies have shown that specific endogenous circRNAs can encode peptides via three mechanisms: 1, by m6A modification to initiate translation: m6A is an adenosine methylation modification of RNA. It is generally considered a key component of circRNA involved in the translation initiation. Modified circRNA is recognized by the YTHDF3 protein, which binds to translation initiation factor eIF4G2, which in turn, recruits eIF4A and eIF4B to form the translation initiation complex and ultimately initiate circRNA translation. In contrast, the m6A demethylase FTO inhibits circRNA translation, and the methyltransferase METTL3/14 increases the translation rate.2, Initiation of translation by IRES: IRESs are common factors that affect circRNA translation. An IRES is an RNA regulatory element that folds into a tRNA-like structure, is recognized by eIF4G2, and recruits ribosomes, thereby initiating direct translation of a protein independent of the 5’ cap structure.3, Rolling loop amplification: An circRNAs sequence, in which the number of total nucleotides is divisible by 3, an infinite open reading frame (iORF) but not stop codon or IRES; therefore, it can be continuously translated produce high molecular-weight proteins. iORF translation is terminated when a -1 ribosome shift produces a stop codon [22, 23]. The crucial functions of these mechanisms in cellular stress response, development, apoptosis, and cell cycle regulation indicate that the products resulting from circRNA translation also exert a significant influence on these biological processes.

### miRNAs

miRNAs are small noncoding RNAs with an average length of 22 nucleotides. miRNAs prevent the translation of messenger RNAs into proteins, thereby inhibiting the expression of specific target genes [24, 25]. Two maturation phases characterize the synthesis of miRNAs: transcription by RNA polymerase II produces pri-miRNAs, which are subsequently processed into pre-miRNAs, which are then transformed into mature miRNAs [26]. miRNAs are essential for normal biological development and are involved in many biological processes [27]. Previously, miRNAs were thought to be ncRNAs and not converted into peptides or proteins. However, increasing evidence is showing that pri-miRNAs carry sORFs that encode regulatory polypeptides, also called miPEPs, which promote the transcription of corresponding miRNAs and function through a novel molecular mechanism. Some miPEPs have been reported to exhibit biological processes in plants [28–30]. These miPEPs boost the transcription of target pri-miRNAs, increasing the concentrations of the associated miRNAs that suppress the expression of target genes in plant cells. Therefore, it has been demonstrated that miPEPs impact miRNA regulatory networks, affecting plant growth and development.

### UTRs

In addition to these aforementioned noncoding RNAs shown to encode micropeptides, sORFs have identified in sequences originally identified as untranslated regions (UTRs), with sORFs in the 5’UTR called upstream (u)ORFs and sORFs in the 3’UTR called downstream (d)ORFs; these uORF/dORFs located in mRNA have also been shown to be translated into micropeptides [12]. Multiple functional micropeptides are encoded by uORFs, and Chen et al. discovered that these micropeptides interact with proteins encoded downstream in the same mRNA sequence [31]. In addition, uORF-encoded peptides play an essential role in tumorigenesis. Huang et al. identified a micropeptide MP31, in mitochondria and that is encoded by the PTEN 5′ UTR, which can inhibit the lactate-pyruvate conversion, and its deletion promotes lactate metabolism and oxidative phosphorylation in glioblastoma cells [32]. Hundreds of dORFs have been in 3’UTRs and to affect classical ORF translation, and dORF encoding, which is not usually conserved, exerts its effect, not through the function of the conserved small peptide, it encodes but through the translational activity of the dORF itself [33]. In conclusion, translatable uORFs/dORFs are widespread in both plants and vertebrates, and are involved in ribosome translation. uORFs commonly repress the translation of downstream CDS regions, while translatable dORFs contribute to the enhanced translation efficiency of the mRNAs that carry them, leading to critical functional repression. These uORFs/dORFs encode micropeptides that exhibit a range of biological functions and that warrant further study.

## Prediction of micropeptide coding potential

Although we have learned that some sORFs show the potential to encode micropeptides, we know little about the function of the vast majority of sORF-encoded micropeptides because distinguishing between coding and noncoding sORFs is not easy. Many methods for identifying proteins used to identify micropeptides are insufficient, and numerous sORFs have unusual start codons that make identifying them even more difficult. However, in recent years, technological advances based on methods such as “histology” have pushed the field forward. Herein, we provide a brief overview of these methods, which can be divided into computer simulation techniques, ribosomal analysis techniques and mass spectrometry techniques.

### Ribosome blotting based sequencing analysis technology

Ribosome sequencing (Ribo-Seq) is a deep sequencing-based tool first described by Ingolia et al. that has been increasingly used to identify potential small peptides in organisms. The process depends on the ability of actively translated ribosomes to prevent nuclease digestion of RNA fragments that are 20–30 nucleotides in length. These ribosome-protected fragments, or “ribosomal footprints,” can be used to map the positions of ribosome sequences. These fragments can also be used to analyse translational activity and identify modifications in translation in response to environmental stress [34, 35]. In recent years, Ribo-Seq and related software have played a crucial role in identifying potential micropeptides in organisms [36–38]. It is important to remember that ribosomal occupancy does not always imply true coding potential or protein function. It has been demonstrated that not all translation events result in stable, functioning peptides. For instance, the H19 mouse transcript is associated with a polypeptide but functions as a ncRNA that controls the translation of the mRNA encoding hormone insulin-like growth factor 2 [39]. Thus, ribosome occupancy is not the only factor used to ascertain whether a ncRNA codes a protein. Recently, many enhanced methods have been developed, and Aspden et al. addressed the complexity of identifying coding from ncRNA by improving the biochemical basis of ribosome analysis. Their technique, known as poly-RIBO-Seq, explicitly enables the detection of transcripts that are actively translated and coupled to multiple ribosomes by taking advantage of the capacity of numerous ribosomes to attach to the same RNA transcript during translation and form a multimer [40]. Moreover, based on ribosome analysis, many databases have been successively created, including SmProt [41], TISdb [42] and sORFs.org [43], which have contributed greatly to the study of micropeptides. In conclusion, with the ongoing advancement of biological technology, proteogenomic approaches combining RNA sequencing, ribosome analysis, and mass spectrometry are critical methods for extending the validity of the data collected and examining the roles of novel micropeptides encoded by ncRNAs.

### Mass spectrometry techniques

By creating, separating, and detecting gas-phase ions, mass spectrometry (MS), a specialized method used for identifying molecules, can be used identify various substances [44]. When using mass spectrometry, charged atoms, molecules, or molecular fragments are arranged according to their mass-to-charge ratio, and corresponding spectral lines are generated. The gold standard for protein identification is mass spectrometry-based protein histology, which can directly evaluate the protein-coding capability of transcripts by detecting peptides translated from sORF [45]. Notably, many MS fragment profiles are unidentifiable through proteomics studies, because, for one reason, some of the features of micropeptides have not yet been annotated. Another drawback of MS-based micropeptide identification is the loss of tiny proteins during sample preparation and purification [6, 46]. Therefore, MS proteomics is frequently employed along with genomic techniques such as RNA sequencing to achieve reliable results for identifying micropeptides [47, 48]. Researchers have characterized micropeptides in several cell lines using proteogenomic or MS-based approaches [49]. Slavoff et al. used proteomics to combine peptidomics with RNA sequencing data to characterize sORFs in K562 cells. Based on the annotated translatome of the human genome, they built a custom database of all sequences with the potential to encode small peptides with more than eight amino acids. They compared their proteomics data to their custom sequencing database and employed liquid chromatography-tandem mass spectrometry (LC-MS/MS) to find sORF-encoded peptides (SEPs) frequently missed using other strategies. Using the aforementioned techniques, they eventually identified 86 previously uncharacterized peptides in K562 cells [49]. In summary, mass spectrometry-based proteomics is a powerful approach that facilitates the discovery and identification of small, uncharacterized proteins. In general, identifying MS-generated small peptides can provide reliable evidence for the presence of SEPs. However, the failure to identify a SEP does not mean that that a Sorf has not been translated. Therefore, in the future, we need to use computational science based on integrated interdisciplinary and experimental studies to characterize small peptides precisely and efficiently.

### Bioinformatics-based technology

Ribosome analysis (Ribo-Seq) and mass spectrometry experiments are currently the most commonly used methods to detect misannotated noncoding RNAs, but these experiments are cell-type dependent and expensive. Therefore, some algorithms have been created to evaluate the coding capability of sORFs derived from ncRNAs. Bioinformatics prediction tools are based on different algorithms and used to examine data and forecast functional unopen reading frames. Coding and noncoding sequences are difficult to distinguish because these transcripts are similar in many respects. However, the low nucleotide sequence conservation of noncoding regions is a crucial distinction, whereas evolutionary conserved protein sequences are distinguishing features of functional sORFs [50]. Therefore, to separate true protein-coding sORFs from noncoding sequences, computational methods based mostly on sequence conservation have been devised. For example, phylogenetic codon substitution frequency (PhyloCSF) analysis is a powerful computational approach. It is used to examine the evolutionary features of nucleotide sequence comparisons to determine the likelihood that a protein-coding region conserved, as indicated by formal statistical comparisons of phylogenetic codon models [51]. Nabi et al. established a system that is based on training dynamics of deep learning models to assess whether a specific lncRNA transcript is likely to carry sequences that code a protein. This framework aids investigators in identifying previously undiscovered proteomes. This approach can be employed for any species that carries a sufficient number of coding and noncoding sequences. It does not require a prelabelled dataset consisting of positively identified but misannotated lncRNAs. In contrast, it is based on Ribo-Seq data, which are used to discern lncRNAs that have been previously misannotated [52]. Of course, although bioinformatics approaches are essential for identifying sORFs, relying on only one technique to predict the coding potential of sORFs in a specific study is insufficient, and other experimental methods should be combined as appropriate to confirm whether these sORFs can be translated into small functional proteins.

## Validation of micropeptide coding and function

We have outlined several ways to discover sORFs. However, translated peptides with evolutionary conserved sequences do not necessarily exhibit a specific biological function. Despite their small size, micropeptides play vital roles in many biological processes, and therefore further experiments are needed to verify and determine whether micropeptides are functional. A targeted antibody can be designed to confirm whether a micropeptide is endogenously expressed in a specific cell type, as antibodies can specifically recognize a protein [6]. Low expression of micropeptides, may make detection challenging, similar to difficulty of identifying other proteins. Developing an epitope for antibody targeting can also be challenging due to the small micropeptide size [31].Therefore, it is not always true that an endogenous micropeptide cannot be detected by an antibody that specifically targets it.

The coding capacity of a sORF can be evaluated via an in vitro translation assay when it is challenging to produce specific antibodies against a micropeptide [53, 54]. The principle is that full-length micropeptide cDNA is cloned into a plasmid containing a T7 or SP6 promoter. Protein translation verifies vector expression in a protein-free environment and in the presence of 35 S-methionine. For detecting the generation of 35 S-methionine-tagged peptides, gel electrophoresis, and autoradiography, which are used to detect proteins expression, can be used discriminate between coding and noncoding transcripts. The results should be interpreted cautiously because sequences can be translated in vitro but not in vivo; however, this technique can be instructive and beneficial for screening potential coding sORFs. On the other hand, a vector that does not produce a stable peptide in vitro may still encode a potential peptide in vivo.

**Fig. 1:**
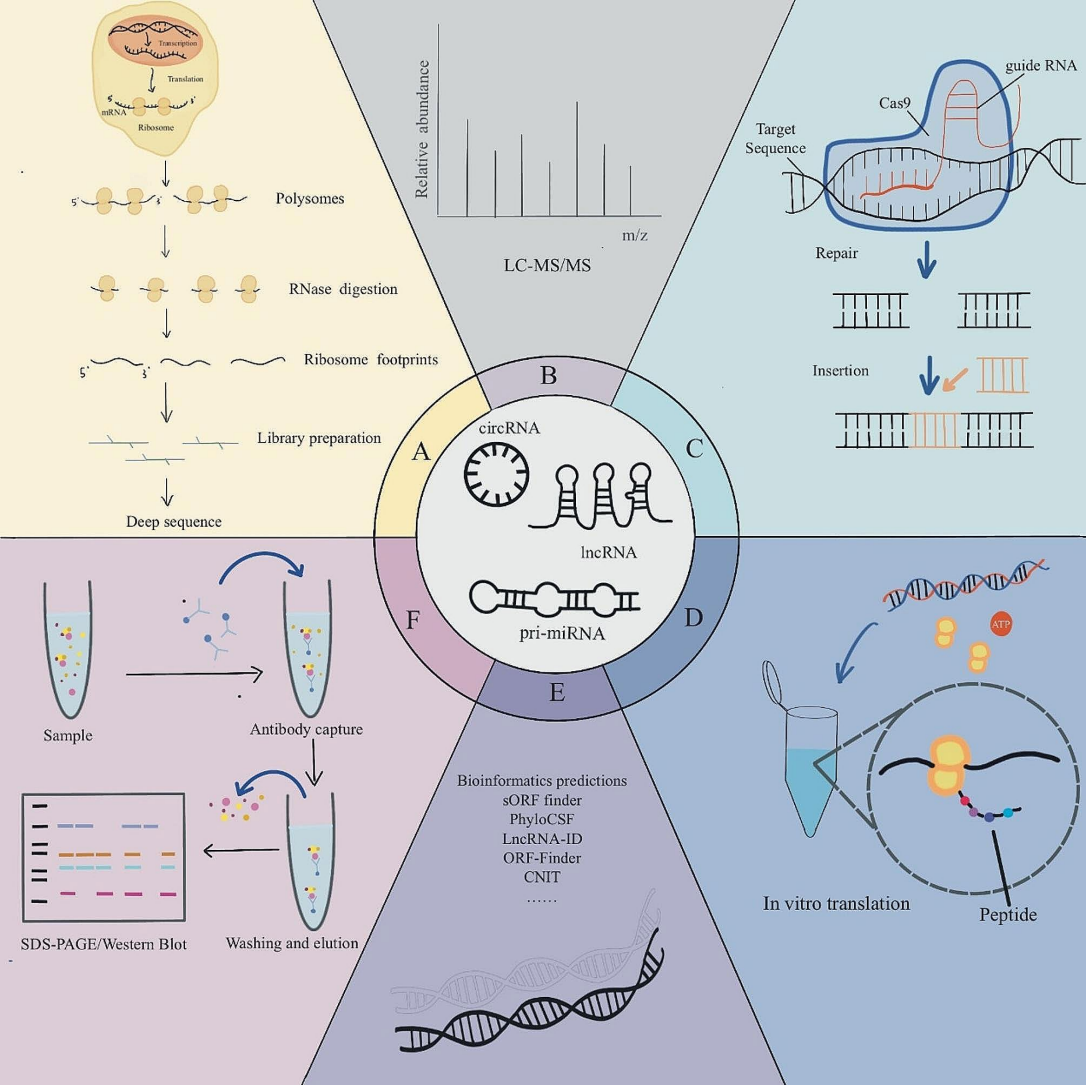
Several techniques for the identification of micropeptides. (**A**) Ribosome sequencing: identifying sORFs with coding potential. (**B**) Liquid chromatography-tandem mass spectrometry (LC-MS/MS) provides reliable evidence for the presence of sORF-encoded peptides (SEPs). (**C**) CRISPR-Cas9: Determining the function of micropeptides by knocking down the sORF of non-canonical peptides. (**D**) In vitro translation: assessing the encoding capacity of Sorf. (**E**) Bioinformatics analysis: algorithmic analysis and database search to obtain potential micropeptides. (**F**) Western Blot:Perform micropeptide coding and functional validation

In addition to the methods described above, CRISPR (Clustered Regularly Interspaced Short Palindromic Repeats)-Cas9 (CRISPR-Associated Protein 9)-mediated gene editing strategies have played an important role in the studies of micropeptide encoding and functional validation [55, 56]. Using CRISPR-Cas9 technology, epitope tag sequences can be inserted into endogenous motifs of the micropeptide of interest. Fusion proteins generated by CRISPR/Cas9 can be detected using protein blot analysis and immunoprecipitation assays [57, 58]. However, it is crucial to take into account tag location (N-terminal, C-terminal, or internal) as well as the size and biochemical characteristics of the amino acids it encodes when developing epitope tag knock-in because in some circumstances, additional peptides or proteins may change the physiological characteristics or impair the function of a micropeptide [59]. These methods offer a thorough processes for finding novel micropeptides that are presumed to be translated by noncoding RNAs. The validation of the coding potential and function of these micropeptides entails numerous experimental procedures. However, not every putative noncoding RNA or the micropeptide it encodes may be useful in every experiment. The CRISPR-Cas9 system might not function well in the intended cell line, or some micropeptides might be too small for MS identification (Table 1). Therefore, the experimental design must be changed appropriately Fig. 1.

**Table 1:** Advantages and disadvantages of micropeptide identification techniques

Techniques	Advantages	Disadvantages	Reference
Ribosome sequencing	Accurate identification of translation start sites	False positives can be produced due to nonspecific binding	
	Enables protein synthesis processes monitoring in vivo	rRNA and tRNA produce Ribo-Seq noise	39,40
	Useful to investigate extension speed, cotranslation processing, etc	Since RNase digests rRNA, it can damage ribosome integrity and cause experimental bias	
Mass spectrometry	Direct verification of the protein-coding potential of transcripts	Small protein can be lost during sample preparation and purification	
		Depends on data from protein databases	45,46
Bioinformatics	A wide range of emerging tools enables, choices to best meet the need	Micropeptides are easy to miss due to their small size	
		Experiments are needed to confirm the translatability of the identified sORFs	51,52
Other experimental verification	Direct verification of micropeptides	The experimental process is tedious	
	Determination of the subcellular location of micropeptides and micropeptide interactions with other macromolecules		6,53,57

## Tumour-associated micropeptides localize to different subcellular organelles

The cell of an organism is a highly ordered structure, and the interior of the cell can be divided into different organelles or cellular regions according to the spatial distribution and functions of these compartments, including the cell membrane, cytoplasm, mitochondria, the endoplasmic reticulum, the nucleus, etc. All subcellular regions cooperate closely with each other to ensure normal physiological activities. Although the distribution of proteins plays a key role in determining their biological functions, the diverse subcellular location of micropeptides in cancer cells are summarized in this section, reflecting their widespread involvement in critical pathways and essential cellular activities in cancer cells.

### Micropeptides that localize to the cell membrane

Cell membranes are important structures to multiple life activities and maintain the homeostasis of the intracellular environment. A large proportion of the smORFs that have been translated carry transmembrane α-helical motifs, suggesting that their function may be related to an organelle membrane [60]. Micropeptides that are concentrated at the cell membrane are crucial to cancer growth. Water channel protein 2 is a protein localized to the cell membrane that assists in the transmembrane transport of water. The micropeptide MIAC interacts with AQP2 and suppresses the production of ITGB4 and SEPT2, which are essential for the actin cytoskeleton and to control cell mobility; moreover, they prevent the development and metastasis of HNSCC tumours [61]. In renal cell carcinoma, MIAC binds to the AQP2 protein, inhibits EREG/EGFR expression, and activates the downstream pathways PI3K/AKT and MAPK to mediate antitumour effects [62]. In addition, MIAC dysregulation is involved in the development of other tumours, suggesting that MIAC may have broad implications in cancer occurrence and development. According to a study by Cao et al., the DLEU1 lncRNA is encoded by two encoding ORFs (ORF1 and ORF8) and can form a pentameric structure with ion channel activity. This pentameric structure increases glioma cell permeability, which in turn increases brain oedema and even increases the risk of cancer cell growth, invasion, and metastasis [63]. The mitogen-activated protein kinase (MAPK) pathway is stimulated by SMIM30, a micropeptide comprising 59 amino acids that is encoded by linc00998; it enables HCC tumour cells to proliferate and metastasize [54]. Notably, SMIM30 expression levels are associated with low survival rates in HCC patients, suggesting that SMIM30 may be a new therapeutic target for HCC and a novel marker for the diagnosis and prognosis of HCC. In addition to these cancer-related micropeptides, other micropeptides, such as Myomixer, localize to cell membranes [64]. The micropeptides that localize to cell membranes may show, enhanced stability and may resist rapid degradation because of their small molecular weight. On the other hand, their biological functions involve regulating more complex systems through protein-protein interactions. To date, only a fraction of membrane micropeptides have been identified. There may be a large number of micropeptides that localize to cell membranes waiting to be discovered. The elucidation of their functions may drive new advances for regulating life activities via membrane micropeptides.

### Micropeptides that localize to the cytoplasm

The functions of micropeptides that localize to the cytoplasm are generally related to the regulation of RNA decay and are involved in protein phosphorylation, etc. Processing bodies (P-bodies) are cytoplasmic ribonucleoprotein (RNP) particles, consisting mainly of translationally repressed mRNA and proteins associated with mRNA decay, suggesting a role in posttranscriptional regulation [65]. The endogenous micropeptide NoBody controls cellular RNA decapitation by interacting with decapitated proteins directly via the EDC4 and DCP1A proteins and localizing to P-bodies [66]. Although its action is not directly related to cancer, NoBody can affect underlying processes in cancer cells by regulating mRNA decay.

In addition, a number of micropeptides are involved in the phosphorylation of other proteins. CIP2A-BP, a micropeptide encoded by LINC00665, binds directly to the tumorigenic gene CIP2A and activates protein phosphatase 2 A (PP2A), which inhibits the PI3K/AKT signalling pathway, leading to a decrease in the expression levels of MMP-2, MMP-9, and Snail. Therefore, it inhibits the metastasis and invasion of triple-negative breast cancer [67]. Additionally, in triple-negative breast cancer, a micropeptide named ASRPS has been identified and shown to regulate the STAT3/VEGF signalling pathway, bind directly to STAT3, inhibits STAT3 phosphorylation, and reduce VEGF expression, thereby inhibiting tumour angiogenesis [58]. In conclusion, CIP2A-BP and ASRPS can be used in vivo as potential anticancer treatments for triple-negative breast cancer. KRASIM, a 99aa micropeptide encoded by the lncRNA NCBP2-AS2, is expressed differently in HCC cells than healthy hepatocytes. Binding and localizing with KRAS protein in the cytoplasm of human HuH-7 hepatoma cells, KRASIM inhibits the growth and proliferation of HCC cells [68]. Moreover, overexpressed KRASIM in HCC cells lowers KRAS protein levels and decreases ERK signalling activity, offering new insights into the mechanisms controlling oncogenic signalling and HCC treatment. AKT3 -174aa, which is produced by circ-AKT3, competes with phosphorylated PDK1 by attaching to it, thereby suppressing the phosphorylation of AKT-thr308, ultimately downregulating PI3K/AKT signalling pathway activity [69]. CircPPP1R12A-73aa is another circRNA-encoded peptide that stimulates the Hippo-YAP signalling pathway to promote colon cancer (CC) cell proliferation, invasion, and migration [21].

There are also micropeptides that localized in the cytoplasm that have been shown to harbour cancer-inhibitory potential. The growth of esophageal squamous cell carcinoma (ESCC) is facilitated by YY1BM, a 21 aa micropeptide encoded by LINC00278, which blocks the connection between YY1 and androgen receptor (AR), reducing eEF2K production through the AR signalling pathway. Intratumoral injection of this purified micropeptide showed therapeutic effects in xenograft models, indicating its potential as a tumour suppressor [70]. In conclusion, whether through their involvement in RNA decay or protein phosphorylation, regulatory micropeptides in different cancer settings can use the regulatory functions of micropeptides to influence the development of tumour disease, and therefore, in the future, we may be able to use the properties of micropeptides to study clinically targeted drugs and ultimately treat tumours by targeting phosphorylation pathways.

### Micropeptides that localize to the endoplasmic reticulum

The endoplasmic reticulum is the site of protein synthesis, processing, and modification in eukaryotic cells and plays a pivotal role in maintaining protein homeostasis. Endoplasmic reticulum stress plays an important role in tumour cell growth, survival, and differentiation and is involved in all aspects of tumour metastasis [71]. APPLE is a micropeptide that localizes to the endoplasmic reticulum and is encoded by the lncRNA ASH1L-AS1. It stimulates PABPC1-elF4G interactions, mRNA recycling, and the assembly of the eIF4F initiation complex, all of which contribute to the development of various subtypes of AML malignancy [72]. FORCP is an ER-localized protein that is highly expressed in highly differentiated CRC cells and whose transcription is controlled by FOXA1. FORCP inhibits basal cell proliferation and induces apoptosis in response to endoplasmic reticulum stress [73].

Dysregulation of Ca^2+^ homeostasis is a recently characterized feature of malignancy and plays an important role in the development and progression of malignancy. In contrast, the endoplasmic reticulum is a major intracellular calcium store that coordinates calcium levels in other organelles (e.g., mitochondria and lysosomes) to regulate calcium homeostasis. Cancer cells express cancer markers after the expression and activity of Ca^2+^ regulators is altered [74]. Sarco-endoplasmic reticulum Ca^2+^ -ATPase (SERCA), a transmembrane pump that transports Ca^2+^ from the cytoplasm to the sarcoplasmic reticulum, plays an important role in the maintenance of Ca^2+^ homeostasis, cell survival and tumour proliferation [75]. MLN, PLN, and SLN function as inhibitors of SERCA in muscle cells, inducing the reuptake of Ca^2+^ into the sarcoplasmic reticulum and muscle relaxation. Conversely, ELN and ALN, two transmembrane micropeptides, function directly as inhibitors of SERCA pump activity in cells in muscle tissue [53, 76]. By replacing and neutralizing the inhibitory peptides PLN, SLN, and MLN, a different micropeptide called DWORF increases SR Ca^2+^ absorption [77]. In summary, noncoding RNA-encoded micropeptides can regulate Ca^2+^ transport by functioning as activators or inhibitors of SERCA, providing potential theoretical support for developing related drugs.

### Micropeptides that localize to endosomes

Endosomes are membrane-encapsulated vesicular structures that are critical for signalling and cellular regulation and play a fundamental role in many diseases and pathogenic states [78]. CASIMO1 is a micropeptide that localizes to endosomes and interacts with squalene epoxidase (SQLE) to influence cell proliferation and cycle progression and regulates lipid droplet accumulation in breast cancer cells [79]. A recognized oncogenic gene in breast cancer, SQLE is a crucial enzyme in the production of cholesterol. MTOR is a serine/threonine kinase that integrates multiple environmental signals and is activated under favourable conditions to promote cell growth and survival. The endosome-localized micropeptide SPAR exerts antitumour effects in the cells of specific cancer types, such as lymphoma, by reducing the amino acid-sensing pathway activated by mTORC1 [80, 81]. In addition to its expression in vivo in humans, a transmembrane micropeptide Hemotin, encoded by a specific smORF, 88aa, located in the early endosomes in Drosophila macrophages, has been shown in Drosophila to regulate endosomal maturation during phagocytosis by inhibiting 14-3-3ζ in synergy with specific phosphatidylinositol (PI) enzymes [82]. Interestingly, Stannin (Snn), as Hemotin homologue in vertebrates, also regulates endosomal maturation by inhibiting 14-3-3ζ-mediated stimulation of PI kinase function. These findings imply that this regulatory system may have remained unchanged throughout evolution.

### Micropeptides that localize to mitochondria

Mitochondria are the power plants in the cell, functioning as primary energy sources. Since alterations in energy metabolism are common features of cancer, mitochondria may be involved in carcinogenesis. In addition, mitochondria play essential roles in other biological processes intrinsically linked to tumorigenesis, such as biosynthesis, signalling, cell differentiation, apoptosis, maintenance of the cell cycle, and control of cell growth [83, 84]. According to recent research, a large number of micropeptides have been found in mitochondria or show a direct connection to their activity. ASAP, encoded by LINC00467, is a micropeptide that localizes to the mitochondrial inner membrane, and is highly expressed in colorectal cancer (CRC) cells or tissues, increasing mitochondrial ATP production and ATP levels throughout the cell. It is also associated with poor prognosis in rectal cancer patients. Another study showed that ASAP enhances ATP binding by interacting with subunits α and γ (ATP5A and ATP5C). The interaction of ASAP with subunits α and γ (ATP5A and ATP5C) enhances the expression of ATP synthase, increasing ATP synthase activity and the mitochondrial oxygen consumption rate, and thus promotes the proliferation of colorectal cancer cells [85]. According to Xiao et al., the micropeptide MPM, which is encoded by LINC00116, was markedly downregulated in human hepatocellular carcinoma tissues, and this decrease was linked to HCC metastasis and recurrence. Additional research has suggested that decreased MPM expression may facilitate hepatocellular carcinoma spread by boosting complex I mitochondrial activity and the NAD+/NADH ratio [86]. PIGBOS is a micropeptide that localize to the outer mitochondrial membrane and interacts with CLCC1, a putative chloride channel in the endoplasmic reticulum. The absence of PIGBOS in cells markedly increases cell sensitivity to UPR signalling and induces apoptosis [87]. Although the molecular mechanism of PIGBOS function has not been described, this protein clearly sensitizes cells to the UPR and drives their apoptosis; therefore, it is reasonable to assume that inhibition of PIGBOS in cancer cells may exert a therapeutic effect.

**Fig. 2:**
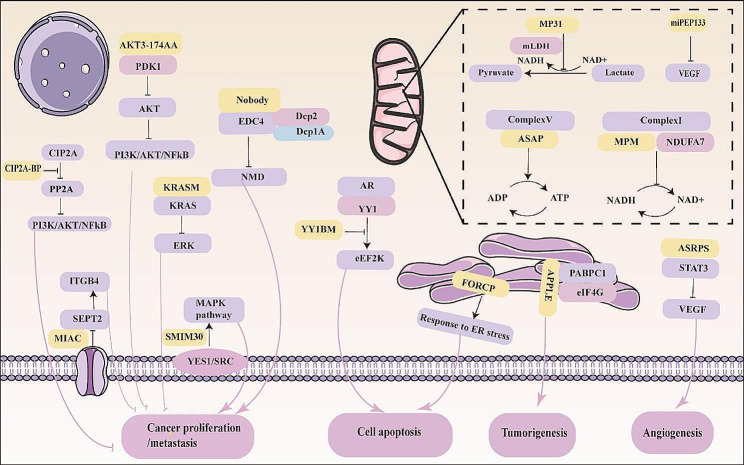
Subcellular localization and mechanism of action of micropeptides in cells. Distribution of micropeptides in cell membrane(SMIM30, MIAC), cytoplasm(CIP2A-BP, YY1BM, AKT3-174AA, KRASM, NoBody, ASRPS), endoplasmic reticulum(FORCP, APPLE), and mitochondria(MP31, miPEP133)

Researchers say that in the presence of cancer cells, mitochondria are driven to unnaturally divide, losing their normal shape and disintegrating around the nucleus, ultimately creating an environment conducive to cancer cell growth [88]. If this process can be blocked, new strategies to arrest tumour growth can be developed. STMP1, a micropeptide of 47 aa localized to the inner mitochondrial membrane, promotes G1/S stage progression and cell proliferation by enhancing the activity of mitochondrial complex IV. In addition, STMP1 upregulates and activates DRP1 in cells of various types of cancer, thereby promoting mitochondrial division and enhancing tumour cell migration [89, 90]. Overall, STMP1 has been identified as a key regulator of tumour metastasis and a novel unit of the mitochondrial division protein machinery. Therefore, it is worthwhile to develop specific inhibitors of STMP1, thereby providing a potential therapeutic target for blocking tumour growth. The tricarboxylic acid (TCA) cycle, a source of energy for cancer, is aided by the mitochondrial conversion of lactate to pyruvate. The micropeptide MP31, which competes with mitochondrial lactate dehydrogenase (mLDH) for nicotinamide adenine dinucleotide (NAD+), limits lactate-pyruvate conversion in mitochondria. It is encoded by the upstream open reading frame (uORF) of homologous phosphatase-tensin (PTEN) [32]. MP31 deficiency promotes lactate metabolism and oxidative phosphorylation (OXPHOS) in glioblastoma, which also suggests that MP31 plays a potential antitumour role. As the sole ATPase in the mitochondrial protein import machinery, HSPA9 controls carcinogenesis, cell proliferation, differentiation, and other processes in addition to impacting mitochondrial function [91, 92]. MiPEP133 is a micropeptide encoded by a primary microRNA, and MiPEP133 is widely distributed in normal human tissues and expressed at low levels in nasopharyngeal carcinoma. By interacting with heat shock protein 70kD (HSPA9) in mitochondria, it obstructs HSPA9’s capacity to engage with its binding partners. This effect reduces both the mitochondrial membrane potential and mass [93]. This study suggests that miPEP133 shows tumour suppressive activity and potential value as a prognostic indicator and therapeutic target in nasopharyngeal carcinoma. In summary, ncRNA-encoded micropeptides can influence tumorigenesis and tumor development by regulating mitochondrial metabolism, oxidative phosphorylation, mitochondrial division, mitochondrial respiratory activity, and the assembly of protein complexes in mitochondria Fig. 2. The discovery of these mitochondrion-localized micropeptides reveals a complex mitochondrial regulatory network and suggests potential targets for the future treatment of tumour diseases.

### Micropeptides that localize to the nucleus

The nucleus is the control centre of the cell and plays an important role in cell metabolism, growth, and differentiation, and is the regulatory centre of intracellular genetics and metabolism. The functions of micropeptides that localize to the nucleus are mainly related to DNA replication, repair, transcription and precursor mRNA splicing. Human CtIP is an 897 amino acid nuclear bridging protein with multiple roles in DNA metabolism and genomic stability. As a tumour suppressor gene and an oncogene with predictive value for tumour progression and therapeutic response, CtIP plays a dual role in cancer [94]. The micropeptide PACMP, which is encoded by lncRNA CTD-2256P15.2, not only inhibits DNA metabolism by inhibiting the association of CtIP with KLHL15 to prevent CtIP ubiquitination, but it also directly binds to DNA damage-induced poly(ADP-ribose) chains to enhance PARP1-dependent poly(ADP-ribose) chemotaxis, functioning in two ways to preserve CtIP [95]. Notably, PACMP is nucleophile-rich and its downregulation can lead to ectopic NPM1 distribution and reduced 47 S pre-rRNA expression, revealing the functional significance of PACMP in maintaining nucleolus homeostasis. Serine and arginine-rich splicing factor 3 (SRSF3) and other splicing regulators interact with lncRNA LOC90024, which encodes SRSP, to control mRNA splicing. Increased SRSP levels are linked to CRC. A malignant phenotype and poor prognosis in CRC patients positively correlate with upregulated SRSP levels [96]. SRSP may be a potential prognostic symptom biomarker and therapeutic target for patients with colorectal cancer.

One of the most frequently activated proto-oncogenes is C-Myc, and chromosomal translocation rearrangements and amplification are two significant factors in the appearance, growth, and evolutionary retreat of tumours in particular tissues [97]. RBRP is a 71 amino acid micropeptide that is encoded by the lncRNA linc00266-1. It exerts an oncogenic effect by binding to the m6A reader IGF2BP1 and improves the recognition of m6A on RNA by IGF2BP1 to increase the stability and production of c-Myc [57]. In colorectal cancer patients, RBRP levels are increased, indicating a more aggressive clinicopathological phenotype and shorter survival time. Thus, the RBRP peptide may be a potential cancer prognostic biomarker and therapeutic target. HOXB-AS3, a micropeptide encoded by lncRNA HOXB-AS3, has been shown to promote cell proliferation in oral squamous cell carcinoma by maintaining the stability of c-Myc mRNA by directly binding to IGF2BP2, and increased expression of HOXB-AS3 is associated with poor prognosis in oral squamous cell carcinoma [98]. FBXW7-185aa, a protein encoded by Circ-FBXW7 with no clear subcellular localization identified to date, reduces the half-life of c-Myc by antagonizing USP28-induced stabilization of c-Myc, thereby impeding the development of malignant gliomas [99].

Heterogeneous nuclear ribonucleoproteins (hnRNPs), the most abundant nuclear proteins in higher eukaryotes, constitute a class of RNA-binding proteins that play important roles in RNA formation and metabolism [100]. In colon cancer, the micropeptide HOXB-AS3 inhibits PKM2 isoform production and reprogrammes glucose metabolism by blocking hnRNP A1-dependent PKM splicing, miR-18a processing, and thus CRC growth [101]. In addition to affecting the development of oral squamous cell carcinoma and colon cancer, HOXB-AS3 has also been shown to be significantly elevated in non-small cell lung cancer tissues and cells, promoting the growth of radiation-induced lung cancer tumours by regulating the PI3K/AKT pathway [102]. In conclusion, HOXB-AS3 may contribute to the development of radiation-induced lung cancer tumours by targeting PI3K/AKT, providing a new perspective on treating radiation-induced lung cancer.

**Fig. 3:**
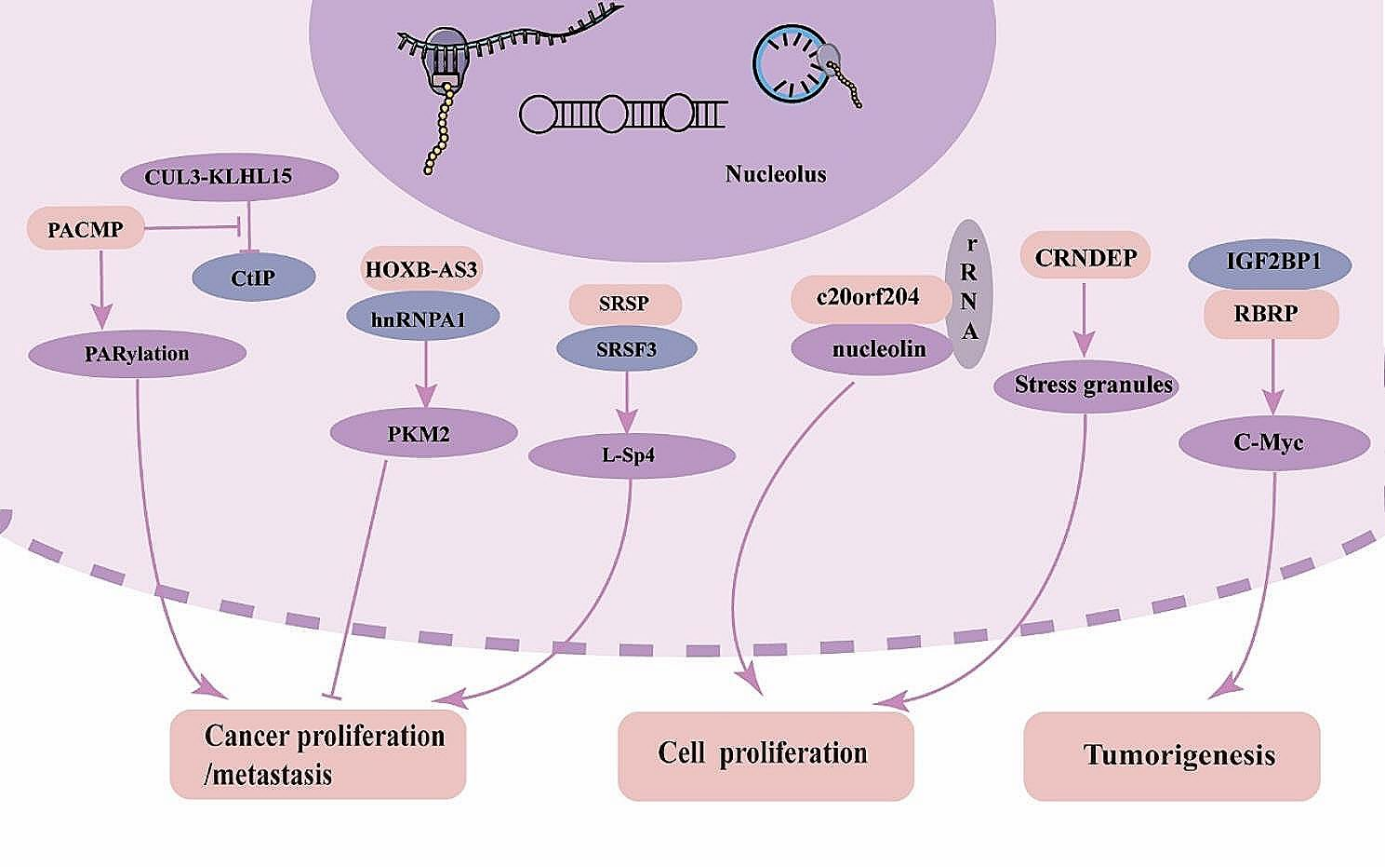
Subcellular localization and mechanism of action of micropeptides in cell nucleus

In addition to the abovementioned micropeptides, there are also micropeptides that localize to the nucleus and influence the progression of cancer. Linc013026-68AA may be used as a target molecule for treating liver cancer because it is expressed in hepatocellular carcinoma cells and contributes to cell growth [103]. CRNDEP, encoded by the lncRNA CRNDE, is highly endogenously expressed in tissues and may be associated with cell proliferation because stress granule formation is increased when CRNDEP is highly expressed [104]. Lu et al. found that overexpression of the lncRNA-encoded novel protein UBAP1-AST6 significantly promoted lung cancer cell proliferation [60]. Nucleolin is associated with multiple steps in ribosome biogenesis, such as RNA pol I synthesis of rRNA and rRNA processing, and direct binding to rRNA. The c20orf204-189AA encoded by lincRNA stabilizes nucleolin and promotes nucleosomal DNA transcription, and overexpression of c20orf204-189AA enhances HCC cell proliferation and nucleosomal DNA transcription, suggesting that this molecule is a cancer-specific micromodulators of HCC formation [105]. CORO1C-47aa is a functional peptide that is encoded by Hsa-circ-0000437. By preventing the connection between ARNT and TACC3, which in turn prevents VEGFA production and secretion and ultimately inhibits angiogenesis, CORO1C-47aa adversely affects tumour angiogenesis [20] (Table 2). The anticancer effect of CORO1C-47aa on EC progression raises the possibility that CORO1C-47aa may be useful in the treatment of tumours, justifying further research Fig. 3.

**Table 2:** The function of ncRNA-encoded micropeptides in human cancer

Subcellular localization	NcRNAs	Micropeptide	Size	Cancer types	Function	Reference
Membrane	LncRNARP11-469H8.6	MIAC	51 aa	HNSCC	Interacts with AQP2 and reduces the expression of ITGB4 and SEPT2, inhibiting tumour growth and metastasis	61
	LINC00998	SMIM30	59 aa	HCC	Activates the MAPK pathway to promote proliferation and metastasis of tumour	54
	lncRNA DLEU1	ORF1,ORF8	-	Glioma	Forms a pentameric structure with ion channel activity, increases brain oedema and the risk of cancer cell development	63
Cytoplasm	LINC00665	CIP2A-BP	52 aa	TNBC	Directly binds to the oncogene CIP2A and inhibits the PI3K/AKT signalling pathway, thus inhibiting the migration and invasion of TNBC.	67
	LINC00278	YY1BM	21 aa	ESCC	Inhibits the interaction of YY1 and androgen receptor and reduces the expression of Eef2k	70
	LINC00908	ASRPS	60 aa	TNBC	Inhibits STAT3 phosphorylation, reduces VEGF expression, and then inhibits tumour angiogenesis	58
	LINC01420	NOBODY	7 kDa	Lung cancer	Affect underlying cancer cells processes by regulating the decay of mRNA	66
	lncRNA NCBP2-AS2	KRASIM	99 aa	HCC	Inhibits the growth and proliferation of HCC cells	68
	Circ-AKT3	AKT3-174aa	174 aa	GBM	Inhibits AKT-thr308 phosphory-lation and PI3K/AKT signalling pathway activation	69
Endoplasmic reticulum	lncRNA ASH1L-AS1	APPLE	90 aa	AML	Promotes the development of different subtypes of AML	72
	LINC00675	FORCP	79 aa	CRC	Inhibits basal cell proliferation and tumorigenesis	73
Endosomes	-	CASIMO1	83 aa	BC	Interacts with squalene epoxidase (SQLE) to influence cell proliferation and cycle progression	79
	lncRNA LINC00961	SPAR	90 aa	Lymphoma	Reduces the effects of amino acid-sensing pathway activated by mTORC1 and exerts antitumour effects	80
Mitochondria	LINC00467	ASAP	94 aa	CRC	Promotes cancer cell proliferation by regulating the production of ATP	85
	LINC00116	MPM	56 aa	HCC	Promotes hepatocellular carcinoma metastasis by increasing mitochondrial complex I activity and the NAD+/NADH ratio	86
	-	PIGBOS	54 aa	-	Regulates the unfolded protein response and cell death	87
	-	STMP1	47 aa	HCC	Promotes G1/S conversion and cell proliferation by enhancing the activity of mitochondrial complex IV	89,90
	5’UTR of PTEN	MP31	31 aa	GBM	Limits lactate-pyruvate conversion in mitochondria	32
	miR-34a	miPEP133	133 aa	NPC	Binds HSPA9 to reduce mitochondrial membrane potential and mitochondrial mass, and inhibits tumour growth	93
Nucleus	lncRNA CTD-2256P15.2	PACMP	44 aa	BC	Maintains nucleolus homeostasis and promotes the DNA damage response	95
	LOC90024	SRSP	130 aa	CRC	Incerases SRSP levels, which are positively correlated with a malignant phenotype and poor prognosis	96
	LINC00266-1	RBRP	71 aa	CRC	Promotes proliferation, colony formation, migration and invasion of cancer cells	57
	lncRNA HOXB-AS3	HOXB-AS3	53 aa	CRC	Inhibits the proliferation, migration and invasion of cancer cells	101
	Linc013026	Linc013026-68AA	68 aa	HCC	Promotes the proliferation of cancer cells	103
	lncRNA	UBAP1-AST6	117 aa	Lung cancer	When overexpressed promotes the proliferation of lung cancer cells	60
	Linc00176 C20orf204	C20orf204-189AA	189 aa	HCC	Stabilizes nucleolin and promotes nucleosomal DNA transcription	105
	circ-0000437	CORO1C-47aa	47 aa	Endometrial cancer	Inhibits endothelial cell proliferation and tumour angiogenesis	20

### Other cancer-related micropeptides with no subcellular location identified

There are also micropeptides, for which a clear specific subcellular localization has not been identified to date, but that also play key regulatory roles in cancer development, suggesting opportunities for using them in the development of cancer markers, drug targets and small-molecule peptide drugs. Dysregulated glutamine metabolism is a distinct metabolic features of cancer cells but not normal cells. Cancer cells consume glutamine (Gln) to generate energy as well as to synthesize other molecules essential for cancer cell growth and progression [106]. The micropeptide XBP1SBM, which is derived from the lncRNA MLLT4-AS1 and highly upregulated in a Gln-deficient environment, prompts the activation of XBP1s and upregulation of VEGF expression by hindering the interaction between XBP1s and XBP1u, thereby augmenting the nuclear localization of XBP1s. These mechanisms ultimately boost the Gln supply and stimulate angiogenesis and metastasis in TNBC cells [107]. PINT87aa, encoded by LINC-PINT, has been found to be overexpressed in senescent hepatocellular carcinoma cells and to arrest the cell cycle, induce cellular senescence and inhibit mitochondrial autophagy, providing evidence for is potential use as a therapeutic target for HCC [108]. Translational growth factor β (TGF-β) is a multifunctional cytokine that is closely linked to cancer pathogenesis [109]. JunBP, a micropeptide encoded by LINC02551, which is upregulated in HCC cells upon TGF-β stimulation, supports hepatocellular carcinoma metastasis by binding to c-Jun to promote its phosphorylation activation [110]. This pathway is a possible HCC treatment target as well as predictive biomarker. The micropeptide AC115619-22aa, encoded by the lncRNA AC115619, can inhibit tumour growth by decreasing the level of m6A modification and thereby inhibiting tumour growth [111]. The micropeptide ACLY-BP, encoded by the lncRNA LINC00887, promotes tumour growth by regulating lipid metabolism and promoting the proliferation of bright cell renal cell carcinoma cells [112]. CTSGDP-13, a novel peptide derived from Cathepsin G, has been found to inhibit bladder cancer development by promoting ferroptosis [113]. In summary, the study of micropeptides can help to develop new diagnostic and therapeutic targets, thus providing a broader perspective for the treatment of tumours.

**Fig. 4:**
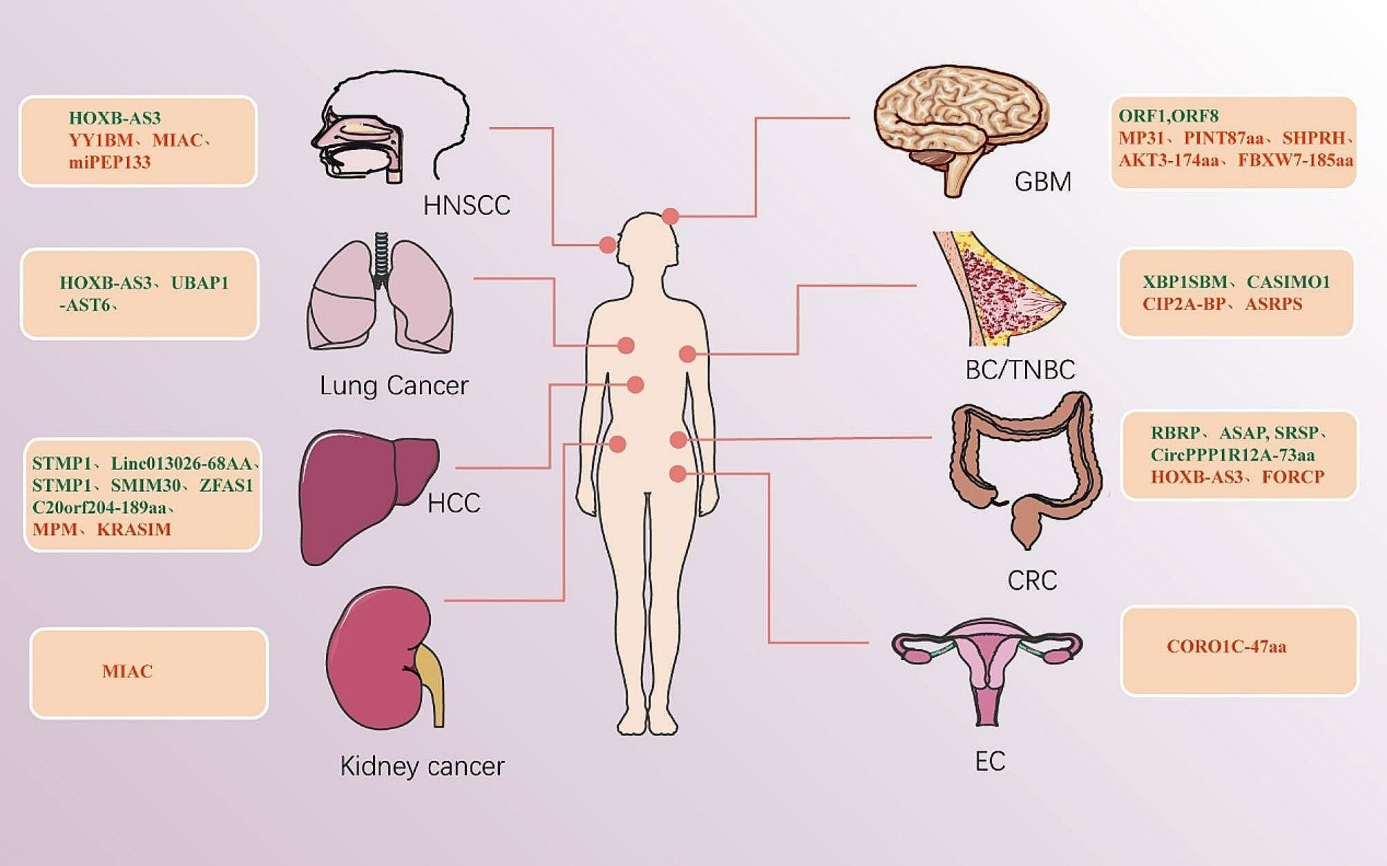
The role of micropeptides in different types of cancer, red indicates tumor suppressor microproteins with potential pharmacological activity, while green indicates cancer-promoting peptides that can be clinically targeted.HNSCC, head and neck squamous cell carcinoma; HCC, hepatocellular carcinoma; GBM, glioblastoma; BC, breast cancer; TNBC, triple-negative breast cancer; CRC, colorectal cancer; EC, endometrial cancer

Interestingly, Jiang et al. found that LINC02381-aa, a micropeptide encoded by LINC02381, localizes to exosomes and may regulate iron concentration in GBM via the action of the glucose transporter SLC2A10 [114]. Furthermore, it was found that exosomal lncAKR1C2 can encode a microprotein with biological functions in recipient cells and promotes the expression of CPT1A by regulating YAP phosphorylation, which leads to enhanced fatty acid oxidation and ATP production. These findings suggest that microproteins encoded by exosomal lncAKR1C2 could serve as therapeutic targets for advanced gastric cancer [115]. To increase the precision and effectiveness of drug therapy, the high biocompatibility of exosomes may be leveraged to develop a delivery system that targets therapeutic factors. Thus, delivery of micropeptides via exosomes is a potential therapeutic strategy Fig. 4.

## Prospects for the application of micropeptides for tumour therapy

### Micropeptides as diagnostic markers of early stage disease and effective therapeutic targets for cancer

The goal of oncology research is to identify additional target molecules for tumour therapy, and to develop tumour vaccines that can be used to prevent tumour occurrence, new technologies that can be used for early tumour diagnosis, and new diagnostic indicators. With the advancement of technology, an increasing number of micropeptides have been identified, some micropeptides such as RBRP and SRSP, are cancere-promoting peptides, while some micropeptides such as KRASIM and ASRPS, are cancer-suppressing peptides. As mentioned above, in TNBC patients, the micropeptide ASRPS inhibited tumor angiogenesis by modulating the STAT3/VEGF pathway, and intratumoral injection of ASRPS also prolonged the survival time in TNBC mouse models. Therefore, ASRPS could be a potential therapeutic target for TNBC [58]. In CRC patients, the upregulation of SRSP levels was significantly associated with poor prognosis and malignancy of the tumor. Therefore, it may be a marker for early diagnosis and a potential therapeutic target in CRC patients [96]. In addition, many micropeptides are also related to the prognosis of tumour patients. Therefore, these micropeptides encoded by small reading frames have potential to be new diagnostic markers for tumours, which may facilitate early diagnosis or improve the efficiency of tumour diagnosis, improve tumour disease monitoring and increase the accuracy of tumour prognosis prediction.

### Potential as a cancer treatment drug

In addition to their great potential in tumour diagnosis and prognosis, micropeptides may also show potential as cancer therapeutic agents due to their small size, high specificity and low cytotoxicity [116]. Micropeptides encoded by ncRNAs play important roles in regulating tumour energy metabolism, c-Myc stability and tumour angiogenesis, suggesting their promise as therapeutic agents for tumours. Of course, micropeptides also have some unavoidable shortcomings, such as short circulating half-life, low affinity, etc. Therefore, in the development of corresponding cancer-targeting drugs, the molecular structure of small peptides can be modified through cyclization, glycosylation, esterification, manipulation of the amino acid order, and polymer splicing and other peptide chain modification methods, so as to enhance the penetration ability of the biofilm, to increase the affinity and metabolic stability of its binding to the receptor, and to further reduce the immunogenicity [117, 118].

In addition, how to deliver micropeptides safely and effectively to target organs and target cells is one of the current challenges. Currently, the main methods applied to micropeptide delivery include: assembling with nano-vectors, recombining with adenoviral vectors and injecting them into patients, and delivering them in conjunction with exosomes, etc. By using these methods, we can realize the slow-release and long-lasting delivery of micropeptides, thus enhancing the specificity and effectiveness of the drugs for cancer treatment. Notably, these micropeptides can also be used in combination with conventional anticancer drugs or with radiotherapy and chemotherapy drugs to enhance the effectiveness of cancer treatment [119–122].

A portion of the micropeptides that have been discovered to date exhibit tissue specificity, making them potentially effective at targeting the disease and limiting the growth and survival of cancer cells without harming healthy cells. They may exert their anticancer effects without negative impact on cellular physiological pathways due to their high specificity for particular cancer cell-specific pathways. Micropeptides are key sources of targetable tumour-specific antigens, showing considerable promise for use in the development of tumour vaccines, and they exhibit great potential as treatments to increase cancer immunogenicity. In addition, studies have shown that targeting lnc15.2/PACMP can suppress tumour resistance by modulating CtIP abundance and PARylation [95]. The expression of CTBP1-dt lncRNA and its encoded microprotein DDUP was negatively correlated with the prognosis of patients with cisplatin-resistant ovarian cancer, which also provides new insights into the treatment of cisplatin-resistant ovarian cancer [123]. MP31 is a micropeptide localised in the mitochondria, it has been found that MP31 disrupts the homeostasis of mitochondria in cancer cells and sensitises GBM cells to chemotherapeutic drugs, therefore, MP31 is promising to be used in the treatment of GBM in the future [124]. Interestingly, it was found that the IRX4-derived micropeptide IRX4_PEP1 promotes the proliferation, migration and invasion of prostate cancer cells by interacting with heterogeneous nuclear ribonucleoprotein K. Not only that, IRX4_PEP1 plays an important role in regulating PCa stem cells and chemotherapy resistance, suggesting its potential as a therapeutic target for PCa [125]. In the future, more micropeptides associated with tumor resistance may be discovered, and targeting these micropeptides could offer new avenues for overcoming tumor resistance in therapy.

## Conclusions

In this review, we describe the origins of micropeptides, review related studies on micropeptide identification techniques, and summarize the functions of tumour-associated micropeptides based on their different subcellular localizations, which may lead to the development of new drugs and biomarkers for future use in tumour therapy and diagnosis. The discovery of micropeptides has enrich and expanded the understanding of the diverse roles played by noncoding RNAs in tumour development. However, only a small fraction of ncRNA-encoded micropeptides have been identified thus far, and although the functions of most micropeptides have been determined, their mechanisms of action in cancer remain unclear and need to be further elucidated. Therefore, micropeptides can be further explored in the following respects: 1, Considering that in tumour cells a variety of intercellular communication mechanisms alter the microenvironment, influence the immune system, and encourage metastasis, potential micropeptide involvement in the local or distant microenvironment, particularly in the communication between tumour cells and stromal cells, merits our attention. Additionally, further understanding of the function of micropeptides in normal cells and their dysregulation in cancer cells may provide important information for further understanding carcinogenesis. 2, Because of the small size and instability of micropeptides, and the difficulty of preparing corresponding antibodies due to a limited number of epitopes, many obstacles to the identification of micropeptides remain, and overcoming these barriers depends the identification of more micropeptide, which requires further updates to the necessary technology. 3, All researchers should think about how to design in vivo and in vitro functional studies experiments to gain insight into the mechanism underlying micropeptide production, expression, and action when the results are not initially obvious.

## Data Availability

Not applicable.

## Abbreviations

ncRNAs: noncoding RNAs
ORFs: open reading frames
lncRNAs: long noncoding RNAs
sORFs: small open reading frames
NMD: nonsense-mediated RNA decay
iORF: infinite open reading frame
UTRs: untranslated regions
Ribo-Seq: Ribosome sequencing
MS: mass spectrometry
LC-MS/MS: liquid chromatography-tandem mass spectrometry
SEPs: sORF-encoded peptides
PhyloCSF: phylogenetic codon substitution frequency
CRISPR-Cas9: Clustered Regularly Interspaced Short Palindromic Repeats- CRISPR-Associated Protein 9
P-bodies: Processing bodies
PP2A: protein phosphatase 2 A
ESCC: esophageal squamous cell carcinoma
AR: androgen receptor
SERCA: Sarco-endoplasmic reticulum Ca^2+^ -ATPase
SQLE: squalene epoxidase
CRC: colorectal cancer
TCA: tricarboxylic acid cycle
mLDH: mitochondrial lactate dehydrogenase
NAD+: nicotinamide adenine dinucleotide
PTEN: phosphatase-tensin
OXPHOS: oxidative phosphorylation
HSPA9: heat shock protein family A member 9
SRSF3: serine and arginine-rich splicing factor 3
hnRNPs: heterogeneous nuclear ribonucleoproteins
Gln: glutamine
TGF-β: Translational growth factorβ
HNSCC: head and neck squamous cell carcinoma
HCC: hepatocellular carcinoma
GBM: glioblastoma
BC: breast cancer
TNBC: triple-negative breast cancer
EC: endometrial cancer
